# Circ-SPG11 knockdown hampers IL-1β-induced osteoarthritis progression via targeting miR-337-3p/ADAMTS5

**DOI:** 10.1186/s13018-021-02526-y

**Published:** 2021-06-17

**Authors:** Yongqiang Liu, Qian Li, Zhida Gao, Fang Lei, Xuefeng Gao

**Affiliations:** Department of Orthopedics, Shijiazhuang People’s Hospital, No. 365 Jianhua South Road, Shijiazhang, Hebei 050000 People’s Republic of China

**Keywords:** Osteoarthritis, Circ-SPG11, MiR-337-3p, ADAMTS5, IL-1β

## Abstract

**Background:**

Osteoarthritis (OA) is responsible for the impotent disability in old people. Circular RNA (circRNA) has been reported to be related to the development of diseases. The lack of research on the role of circRNA spastic paraplegia 11 (circ-SPG11) results in conducting this study.

**Methods:**

The expression of circ-SPG11, microRNA-337-3p (miR-337-3p), and aggrecanases like a disintegrin and metalloproteinase with thrombospondin motifs 5 (ADAMTS5) mRNA was detected by quantitative real-time polymerase chain reaction (qRT-PCR). Western blot was used to measure the protein expression of extracellular matrix (ECM) degradation-related markers and ADAMTS5. Ribonuclease R (RNase R) was applied to test the stability of circ-SPG11 in CHON-001 cells. The viability, apoptosis, TNF-α and IL-6 production were determined by cell counting kit-8 (CCK-8) assay, flow cytometry assay, and enzyme-linked immunosorbent assay (ELISA), respectively. Meanwhile, the interaction between miR-337-3p and circ-SPG11 or ADAMTS5 was respectively predicted by Circinteractome or Starbase2.0, which was further verified by dual-luciferase reporter system and RNA binding protein immunoprecipitation (RIP) assay.

**Results:**

Circ-SPG11 and ADAMTS5 were upregulated and miR-337-3p was downregulated in OA tissues and OA model cells. Circ-SPG11 knockdown allayed interleukin 1β (IL-1β)-induced restraint in viability and promotion in apoptosis, TNF-α, and IL-6 generation and ECM degradation in CHON-001 cells. Anti-miR-337-3p or ADAMTS5 overexpression correspondingly reversed si-circ-SPG11 or miR-337-3p overexpression-mediated facilitation in viability, and inhibition in apoptosis, TNF-α and IL-6 generation and ECM degradation in OA model cells. Moreover, anti-miR-337-3p ameliorated si-circ-SPG11-mediated inhibition in ADAMTS5 mRNA and protein expression in OA model cells.

**Conclusion:**

Circ-SPG11 facilitated OA development via regulating miR-337-3p/ADAMTS5 axis. This finding might contribute to the improvement of OA therapy.

## Highlights

1. Circ-SPG11 was upregulated in OA tissues and OA model cells.
2. Circ-SPG11 knockdown facilitated the proliferation and suppressed the apoptosis, inflammatory factors generation, and ECM degradation in OA model cells.
3. Circ-SPG11/miR-337-3p/ADAMTS5 axis stimulated OA development.

## Introduction

As a degenerative arthropathy, osteoarthritis (OA) extensively occurs in the aged including 10% males and 18% females [1]. The irreversible OA progression eventually results in a loss of function of sufferers, thus imposes a heavy burden on mentality and physiology [2]. OA is characterized by the progressive damage in the structure and function of articular subassemblies, especially in cartilage [3]. The disequilibrium between anabolism and catabolism of extracellular matrix (ECM) is the main cause of cartilage damage, which further contributes to the deterioration of mechanical properties [4]. ECM degradation initially occurs in the surface of cartilage with the variation of matrix-degrading enzymes including collagenases metalloproteinases 13 (MMP13) and aggrecan-degrading enzymes (Aggrecan), and calcification in cartilage area gradually forms with the prolongation of degradation time [5]. In the process of self-healing, chondrocytes lead to the reaction of pro-inflammatory including the generation of interleukin 1β (IL-1β), tumor necrosis factor α (TNF-α), and interleukin 6 (IL-6), and stimulate the proliferation of synovial cells which further release pro-inflammatory products [6]. The only effective treatment for arthritis is surgery; however, such costly operation only marginally improves the quality of life of OA patients [7]. Thus, the precision therapy which could alleviate the suffering of OA patients is appealed, and this needs to further understand the regulatory mechanism of OA.

As a novel non-coding RNA, circular RNA (circRNA) has been repeatedly documented to be associated with various diseases. The unique circular structure that formed by the exons, introns, or intergenic regions endows a stable status to circRNA in cells [8]. The endogenous competitive role of circRNA to micro RNA (miRNA) in diseases becomes even more common in numerous reports. For instance, circ-TTBK2 sponged miR-761 to regulate the metastasis and proliferation in glioma [9]. Circ_0076305 harbored miR-296-5p to regulate STAT3 expression, thus participated in the development of non-small cell lung cancer [10]. In terms of the OA, circRNA-9119 protected against apoptosis by sponging miR-26a to regulate PTEN [11]. CircRNA-UBE2G1 acted as a competing endogenous RNA (ceRNA) to interact with miR-373/HIF-1a to deteriorate lipopolysaccharide-induced OA [12]. CircRNA_Atp9b harbored miR-138-5p to advance OA development [13]. There was no consensus on the role of circRNA in OA; thus, more researches are needed to perfect the theory about the regulatory pathway of circRNA in OA. However, we could hardly find any reports about the role of circRNA spastic paraplegia 11 (circ-SPG11) in OA through consulting literature materials, which resulted in setting out to determine the functional mechanism of circ-SPG11 in OA.

In this study, the dysregulation of circ-SPG11 was widely presented in OA tissues and IL-1β-induced OA model cells. Circ-SPG11 deletion or miR-337-3p overexpression resulted in facilitating the proliferation and inhibiting the apoptosis, inflammatory cytokines secretion, and ECM degradation, which was correspondingly reversed by miR-337-3p inhibition or aggrecanases like a disintegrin and metalloproteinase with thrombospondin motifs 5 (ADAMTS5) overexpression in OA model cells. Moreover, circ-SPG11/miR-337-3p/ADAMTS5 axis regulated IL-1β-induced OA development, which might bring novel targeted therapy sites for OA treatment.

## Materials and methods

### Patients and sample collection

All the experiments involved in this study have been approved by the Ethics Committee of Shijiazhuang People’s Hospital. Informed consent has been signed by participants or their legal guardians. The tissue samples used in this experiment were obtained from 29 OA patients who suffered total knee arthroplasty, and 23 non-OA patients who unfortunately suffered a traumatic amputation in Shijiazhuang People’s Hospital. The surgically separated cartilage tissues were promptly saved in liquid nitrogen for further testing.

### Cell culture and treatment

Human chondrogenic cell line CHON-001 was obtained from American Type Culture Collection (ATCC, Rockville, MD, USA). Cells were cultured in Dulbecco’s modified Eagle’s medium (DMEM, Gibco, Carlsbad, CA, USA) supplemented with 10% fetal bovine serum (FBS; Gibco) at 37 °C with 5% CO_2_. For the establishment of OA cellular model in vitro, cells were firstly treated with IL-1β (Sigma-Aldrich, St. Louis, MO, USA) at the concentrations of 0 ng/mL, 5 ng/mL, and 10 ng/mL, 15 ng/mL for 24 h. Then, 10 ng/mL of 1L-1β was chose to further stimulate cells for 0 h, 12 h, 24 h, and 48 h, respectively.

### Cell transfection

Small interfering RNA (siRNA) targeting circ-SPG11 (si-circ-SPG11), circ-SPG11 overexpression (circ-SPG11), miR-337-3p overexpression (miR-337-3p), miR-337-3p inhibition (anti-miR-337-3p), ADAMTS5 overexpression (ADAMTS5), and the corresponding negative controls (si-NC, vector, miR-NC, anti-miR-NC, pcDNA) were purchased from Ribobio (Guangzhou, China). Lipofectomine 3000 transfection reagent (Invitrogen, Carlsbad, CA, USA) was used to perform the transient transfection just following the instruction.

### RNA isolation, ribonuclease R (RNase R) treatment and quantitative real-time polymerase chain reaction (qRT-PCR)

For the isolation of total RNA from CHON-001 cells, TRIzol reagent (Thermo Fisher Scientific, Waltham, MA, USA) was used. For the RNase R treatment, 1 μg total RNA was devoted to incubating with 1 μL RNase R (Epicenter, Madison, WI, USA) at 37 °C for 20 min. For qRT-PCR, the depurative RNA was reverse transcribed into cDNA via applying cDNA reverse transcription kit (Applied Biosystems, Foster City, CA, USA). Then, cDNAs were used as templates for the amplification of the interested genes, which were accomplished by using Prime Script™ RT reagent kit (Takara, Shiga, Japan). Following, the detection of the interested genes expression was realized by Platinum SYBR Green qPCR SuperMix UDG (Invitrogen). GAPDH was used as an internal reference to normalize the expression of circ-SPG11, Linear SPG11, and ADAMTS5, and U6 was used as an internal reference to normalize the expression of miR-337-3p. The 2^−ΔΔCt^ method was applied to calculate relative expression. Primers used in this study were showed as (5′-3′): Circ-SPG11, Forward (F), 5′-AGCTATAGGAGATCCCAAACTG-3′, Reverse (R), 5′-TGGCCTGATTGGTGGGAATC-3′; SPG11, F, 5′-AGTCCACAGCAGACATAAGCAG-3′, R, 5′-CCAATGTGCGGTCTTGACAC-3′; miR-337-3p, F, 5′-CGCGCTCCTATATGATGCCT-3′, R, 5′-AGTGCAGGGTCCGAGGTATT-3′; ADAMTS5, F, 5′-TATGTTCTCCAGAGCGCAGC-3′, R, 5′-GGAATCGTCATGGGAGAGGC-3′; U6, F, 5′-CTCGCTTCGGCAGCACATATACT-3′, R, 5′-ACGCTTCACGAATTTGCGTGTC-3′; GAPDH, F, 5′-GGAGTCCACTGGCGTCTTCA-3′, R, 5′-GGTTCACACCCATGACGAAC-3′.

### Cell counting kit-8 (CCK-8) assay

CCK8 assay was applied to measure the viability of CHON-001 cells. Cell were seeded into 96-well plates and then incubated with CCK-8 solution (Beyotime, Shanghai, China) at 37 °C for 2 h. MultiMode Microplate Reader (Thermo Fisher Scientific) was applied to collect and analyze the signal about the absorbance at 450 nm.

### Flow cytometry assay

The apoptosis rate of CHON-001 cells was measured by Annexin V-Fluorescein isothiocyanate (FITC)/Propidium lodide (PI) (BD Biosciences, San Jose, CA, USA) according to the protocol. Briefly, CHON-001 cells were collected and treated with or without 10 ng/mL of 1L-1β after transient transfection. Afterwards, cells were incubated with Annexin V-FITC and PI for 20 min. A flow cytometry (BD Biosciences) was used to detect the apoptosis.

### Enzyme-linked immunosorbent assay (ELISA)

The supernatant of CHON-001 cells culture was isolated and collected. The production of TNF-α and IL-6 in the supernatant was measured by ELISA kits (RayBiotech, Peachtree Corners, GA, USA) following the manufacture’s protocol.

### Western blot

The total protein of CHON-001 cells was isolated by RIPA buffer (Beyotime). Then, the crude extracted proteins were further subdivided by sodium dodecyl sulfate polyacrylamidegel electrophoresis (SDS-PAGE, Beyotime) and polyvinylidene difluoride membranes (PVDF, Millipore, Danvers, MA, USA). Following, the membranes were blocked by non-fat milk and washed by 1 × Tris-buffered saline tween-20 (TBST, Sigma-Aldrich). Afterwards, the membranes containing the proteins were incubated with the primary antibodies, including anti-MMP13 (1: 3,000, Abcam, Cambridge, UK), anti-ADAMTS5 (1: 250, Abcam), anti-GAPDH (1: 2,500, Abcam), and anti-Aggrecan (1: 100, Abcam), and then incubated with the second antibodies anti-rabbit IgG (1: 20,000, Abcam) or anti-mouse IgG (1: 2,000, Abcam). The result was detected by Electrochemiluminescence (ECL) detection kit (Roche Diagnostics GmbH, Mannheim, Germany).

### Bioinformatic analysis and dual-luciferase reporter system

The combination between miR-337-3p and circ-SPG11 or ADAMTS5 was predicted by Circinteractome and Starbase2.0. The reporter plasmids (pmirGLO, Promega, Madison, WI, USA) containing the potential binding sequences or corresponding mutated sequences between miR-337-3p and circ-SPG11 or ADAMTS5, which were named as WT-circ-SPG11, WT-ADAMTS5 3′ untranslated regions (UTR) or MUT-circ-SPG11, MUT-ADAMTS5 3′UTR, were transfected with miR-337-3p or miR-NC into CHON-001 cells by using Lipofectamine3000 (Invitrogen). The luciferase activity was detected by Dual-Luciferase® Reporter Assay System (Promega) following the instruction.

### RNA binding protein immunoprecipitation (RIP) assay

Magna RNA-binding protein immunoprecipitation kit (Merck KGaA, Darmstadt, Germany) was devoted to performing RIP assay. Prior to conduct the RIP assay, CHON-001 cells were lysed by RIP Lysis Buffer (Beyotime). Then, the lysates were incubated with magnetic beads which were conjugated with Argonaute-2 (Ago2, Millipore) or control immunoglobulin G (IgG, Millipore). The complex was digested by proteinase K (Sigma-Aldrich) and the binding RNAs were identified by qRT-PCR.

### Data analysis

Data used in this study were obtained from three replicates and presented as mean ± standard deviation (SD). The statistical analysis was performed by GraphPad Prism version 7.0 (GraphPad Inc., San Diego, CA, USA). The difference between two or more groups was compared by Student’s *t* test or one-way analysis of variance (ANOVA). Pearson correlation coefficient analysis was conducted to analyze the relationship between the expression of miR-337-3p and circ-SPG11 or ADAMTS5 in OA tissues. *P* < 0.05 was determined as statistical significance.

## Results

### Circ-SPG11 was upregulated in OA tissues and OA model cells

**Fig. 1:**
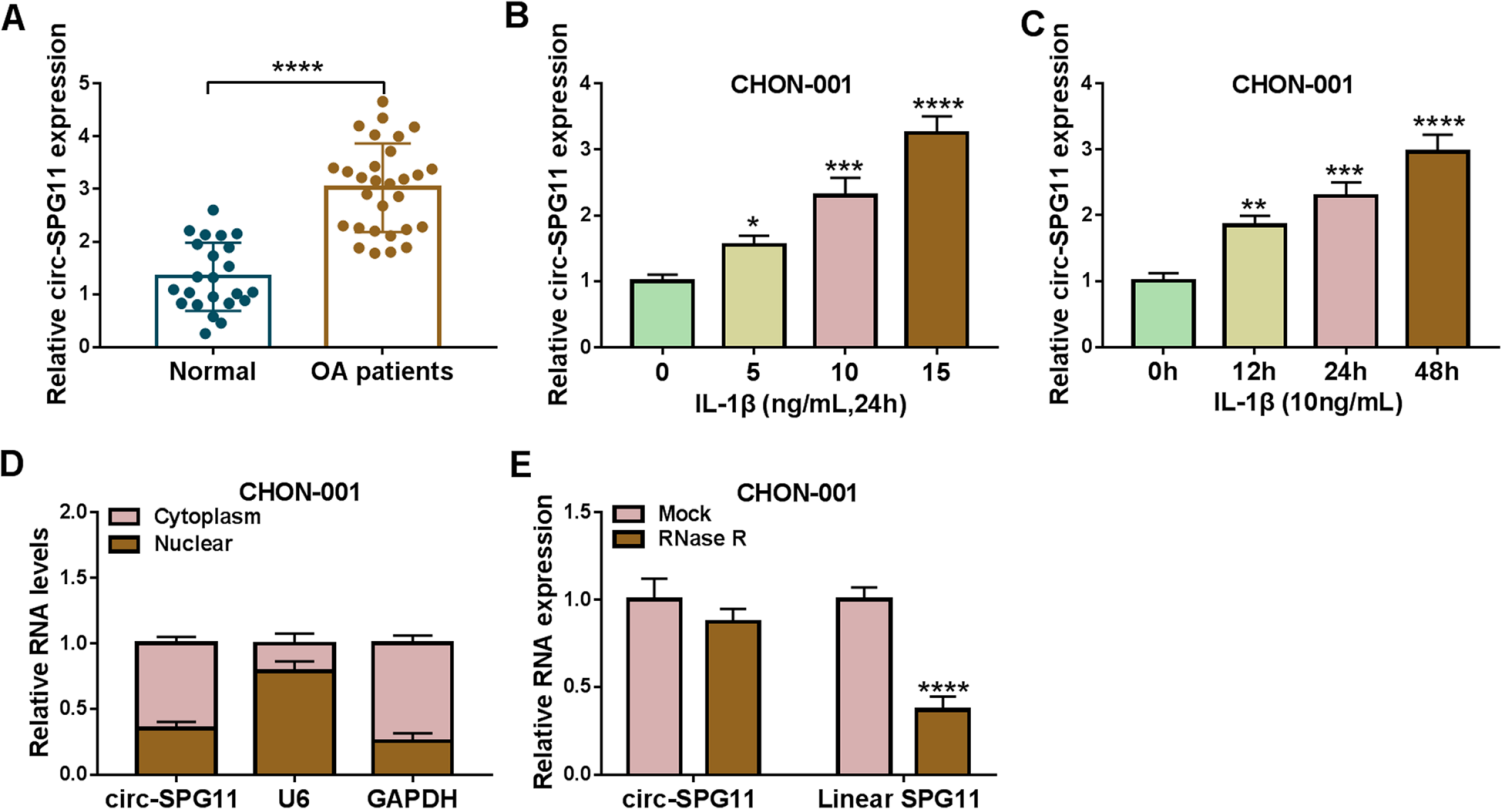
IL-1β treatment triggered the upregulation of circ-SPG11 in CHON-001 cells. **A** Circ-SPG11 expression was detected by qRT-PCR in OA tissues (n = 29) and normal tissues (n = 23). **B** Circ-SPG11 expression was examined by qRT-PCR in CHON-001 cells treated by 0 ng/mL, 5 ng/mL, and 10 ng/mL, 15 ng/mL of IL-1β for 24 h. **C** Circ-SPG11 expression was measured by qRT-PCR in CHON-001 cells treated by 10 ng/mL of IL-1β for 0 h, 12 h, 24 h, and 48 h. **D** QRT-PCR was devoted to testing the expression of circ-SPG11, U6, and GAPDH in the cytoplasm and nuclear of CHON-001 cells. **E** QRT-PCR was used to determine the expression of circ-SPG11 and Linear SPG11 in CHON-001 cells treated by RNase R. *P < 0.05, **P < 0.01, ***P < 0.001, ****P < 0.0001

Due to a supply gap about researching the role of circ-SPG11 in OA, the expression of circ-SPG11 in OA tissues was primarily explored. Circ-SPG11 was significantly upregulated in OA tissues compared with normal tissues (Fig. 1A). In order to further complete the experimental exploration, IL-1β was applied to establish an OA model cells by treating CHON-001 cells with different concentrations of IL-1β or different times. Obviously, IL-1β treatment resulted in dose-dependent and time-dependent increases in circ-SPG11 expression in CHON-001 cells (Fig. 1B, C). Interestingly, circ-SPG11 was mainly distributed in cytoplasm rather than nuclear (Fig. 1D). Meanwhile, the stronger tolerance on RNase R indicated that circ-SPG11 possessed a stable circular structure when compared with Linear SPG11 (Fig. 1E). Taken together, circ-SPG11, which was located primarily in the cytoplasm, was upregulated in OA tissues and IL-1β-induced OA model cells.

### IL-1β inhibited cell viability and induced apoptosis in CHON-001 cells

**Fig. 2:**
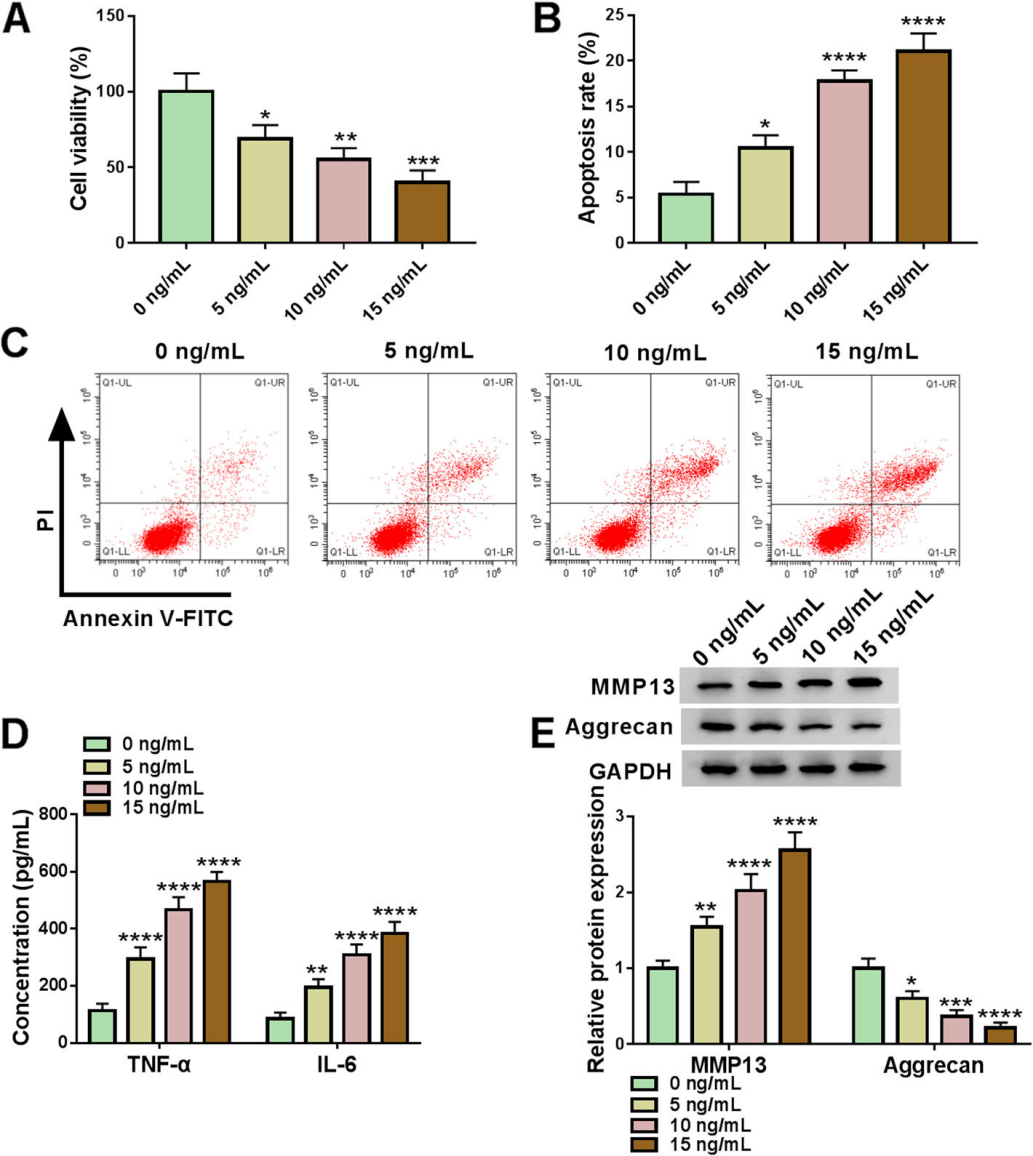
IL-1β regulated CHON-001 cell proliferation, apoptosis, inflammatory cytokines secretion, and ECM degradation. **A** The cell viability was detected by CCK-8 assay. **B**, **C** The flow cytometry assay was assessed to measure the cell apoptosis in CHON-001 cells. **D** The concentration of inflammatory factors TNF-α and IL-6 was detected by ELISA. **E** The western blot assay was utilized to analyze the MMP13 and Aggrecan expressions. *P < 0.05, **P < 0.01, ***P < 0.001, ****P < 0.0001

In order to detect the effects of IL-1β on CHON-001 cells, the cell viability was detected using CCK-8 assay. The data indicated that the cell viability was significantly decreased under different concentrations of IL-1β (5 ng/mL, 10 ng/mL, and 15 ng/mL) (Fig. 2A). In addition, with the increase of IL-1β concentration, the cell apoptosis rate was upregulated (Fig. 2B, C). The secretion of inflammatory factors (TNF-α and IL-6) was markedly increased in CHON-001 cells induced by IL-1β (Fig. 2D). ECM degradation-related marker MMP13 was upregulated and Aggrecan was downregulated with the treatment with IL-1β in CHON-001 cells (Fig. 2E). These results demonstrated that IL-1β inhibited cell viability and promoted cell apoptosis, TNF-α and IL-6 generation, and ECM degradation with the increase of ox-LDL concentrations.

### Circ-SPG11 deletion allayed IL-1β-induced suppression in proliferation and facilitation in apoptosis, inflammatory cytokines secretion, and ECM degradation in CHON-001 cells

**Fig. 3:**
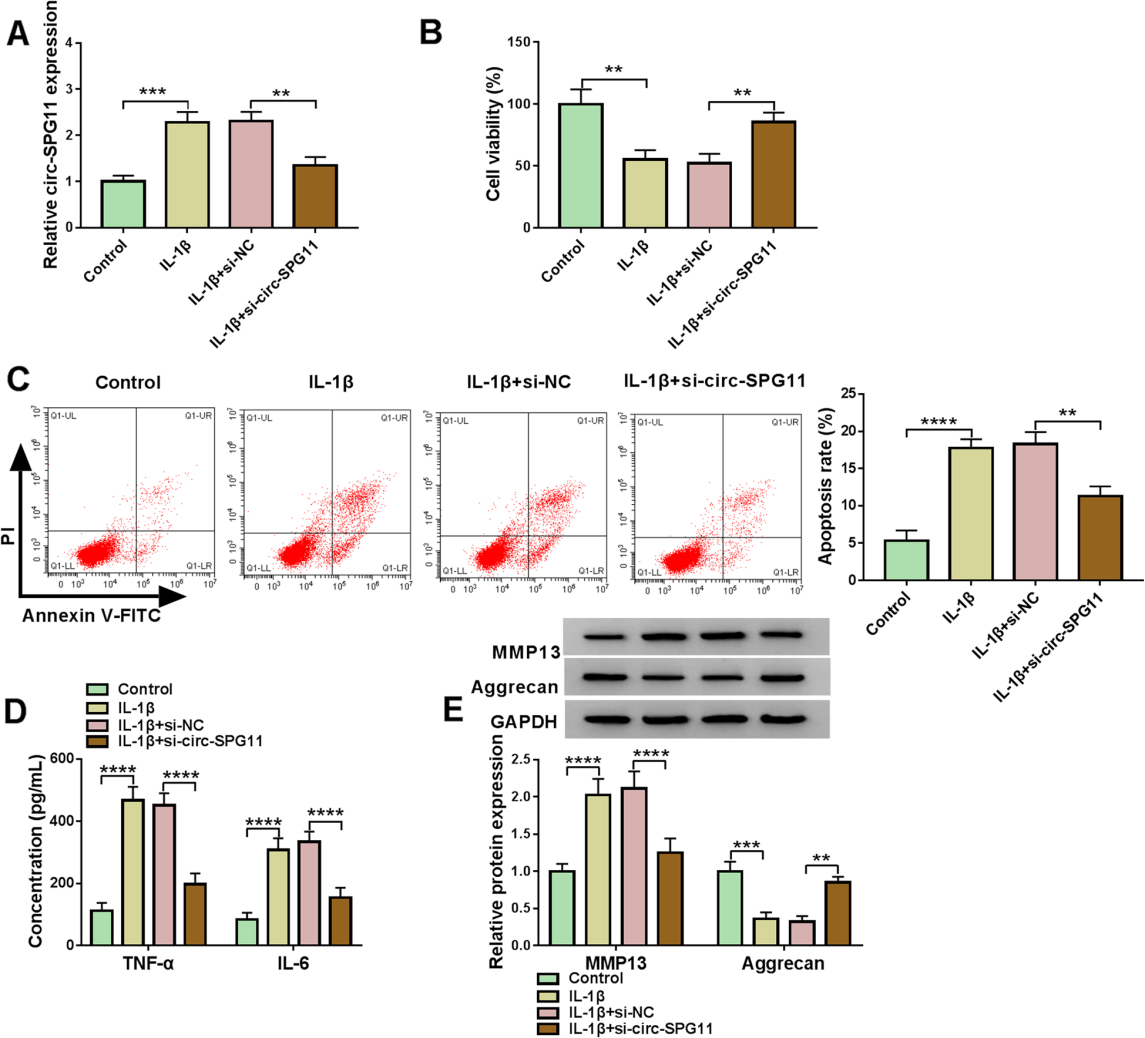
Circ-SPG11 knockdown reversed IL-1β-induced repression in proliferation, stimulation in apoptosis, inflammatory cytokines secretion, and ECM degradation in CHON-001 cells. **A** QRT-PCR was employed to test circ-SPG11 expression in CHON-001 cells without treatment or treated with IL-1β and transfected with si-circ-SPG11 or negative control. **B** CCK8 assay was used to assess the viability in CHON-001 cells without treatment or treated with IL-1β and transfected with si-circ-SPG11 or negative control. **C** Flow cytometry assay was devoted to measuring the apoptosis in CHON-001 cells without treatment or treated with IL-1β and transfected with si-circ-SPG11 or negative control. **D** ELISA was conducted to detect the production of TNF-α and IL-6 in CHON-001 cells without treatment or treated with IL-1β and transfected with si-circ-SPG11 or negative control. **E** Western blot was used to determine the protein expression of MMP13 and Aggrecan in CHON-001 cells without treatment or treated with IL-1β and transfected with si-circ-SPG11 or negative control. **P < 0.01, ***P < 0.001, ****P < 0.0001

To examine the functional role of circ-SPG11 in OA, loss-of-function approaches were designed. As expected, IL-1β treatment resulted in a noticeable increase in circ-SPG11 expression, which was eliminated by circ-SPG11 deletion in CHON-001 cells (Fig. 3A). The viability of CHON-001 cells was restrained by IL-1β; however, this restraint was further reversed by circ-SPG11 deletion (Fig. 3B). IL-1β-mediated promotion in apoptosis rate was ameliorated by circ-SPG11 deletion in CHON-001 cells (Fig. 3C). The secretion of TNF-α and IL-6 was motivated by IL-1β treatment, which was restored by circ-SPG11 deletion in CHON-001 cells (Fig. 3D). In terms of ECM degradation, the promotion in MMP13 protein expression and the inhibition in Aggrecan protein expression were induced by IL-1β treatment; however, the effects of IL-1β on ECM degradation-related markers protein expression were abolished by circ-SPG11 deletion in CHON-001 cells (Fig. 3E). These data proposed that circ-SPG11 knockdown alleviated OA development.

### Circ-SPG11 sponged miR-337-3p

**Fig. 4:**
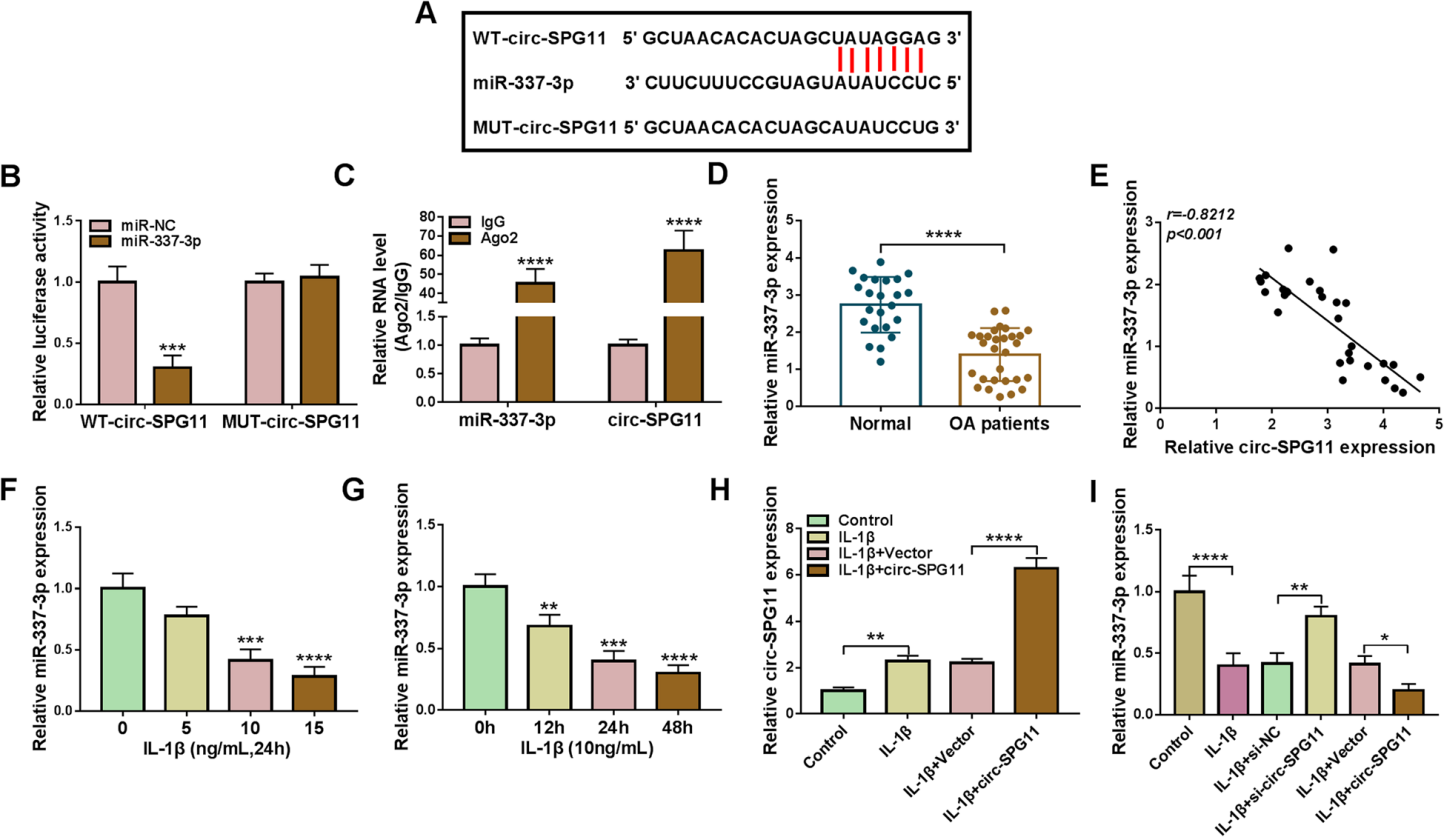
Circ-SPG11 sponged miR-337-3p and negatively regulated miR-337-3p expression. **A** Circinteractome was used to predict the underlying binding sites between circ-SPG11 and miR-337-3p. **B**, **C** Dual-luciferase reporter system and RIP assay were employed to assess the combination between circ-SPG11 and miR-337-3p. **D** QRT-PCR was devoted to testing miR-337-3p expression in OA tissues and normal tissues. **E** The relationship between the expression of circ-SPG11 and miR-337-3p was analyzed by Pearson correlation coefficient. **F** MiR-337-3p expression was examined by qRT-PCR in CHON-001 cells treated by 0 ng/mL, 5 ng/mL, and 10 ng/mL, 15 ng/mL of IL-1β for 24 h. **G** MiR-337-3p expression was examined by qRT-PCR in CHON-001 cells treated by 10 ng/mL of IL-1β for 0 h, 12 h, 24 h, and 48 h. **H** QRT-PCR was used to examine circ-SPG11 expression in CHON-001 cells without treatment or treated with IL-1β and transfected with circ-SPG11 or negative control. **I** MiR-337-3p expression was detected by qRT-PCR in CHON-001 cells without treatment or treated with IL-1β and transfected with circ-SPG11 or negative control. *P < 0.05, **P < 0.01, ***P < 0.001, ****P < 0.0001

The potential regulatory mechanism of circ-SPG11 in OA was further studied. The underlying binding sites between circ-SPG11 and miR-337-3p, which were predicted by Circinteractome online tool, and together with the corresponding mutated sites were presented in Fig. 4A. Subsequently, the dual-luciferase reporter system presented a striking decrease in luciferase activity in CHON-001 cells transfected with miR-337-3p and WT-circ-SPG11, which highlighted the existence of the combination between circ-SPG11 and miR-337-3p in CHON-001 cells (Fig. 4B). Moreover, the marked increase in the level of miR-337-3p and circ-SPG11, which was presented by RIP assay, indicated a direct combination between circ-SPG11 and miR-337-3p (Fig. 4C). MiR-337-3p was strikingly lowly expressed in OA tissues (Fig. 4D). Logically, miR-337-3p expression was negatively correlated with circ-SPG11 expression (Fig. 4E). Interestingly, the decrease in miR-337-3p expression showed a dose-dependent manner and a time-dependent manner to the treatment of IL-1β in CHON-001 cells (Fig. 4F, G). In addition, circ-SPG11 overexpression further boosted IL-1β-induced upregulation in circ-SPG11 expression in CHON-001 cells (Fig. 4H). However, circ-SPG11 knockdown reversed IL-1β-induced inhibition in miR-337-3p expression and circ-SPG11 overexpression further enhanced IL-1β-induced inhibition in miR-337-3p expression in CHON-001 cells (Fig. 4I). These data illustrated that circ-SPG11 harbored miR-337-3p and negatively regulated miR-337-3p expression in IL-1β-treated CHON-001 cells.

### Circ-SPG11 knockdown impeded OA development by sponging miR-337-3p

**Fig. 5:**
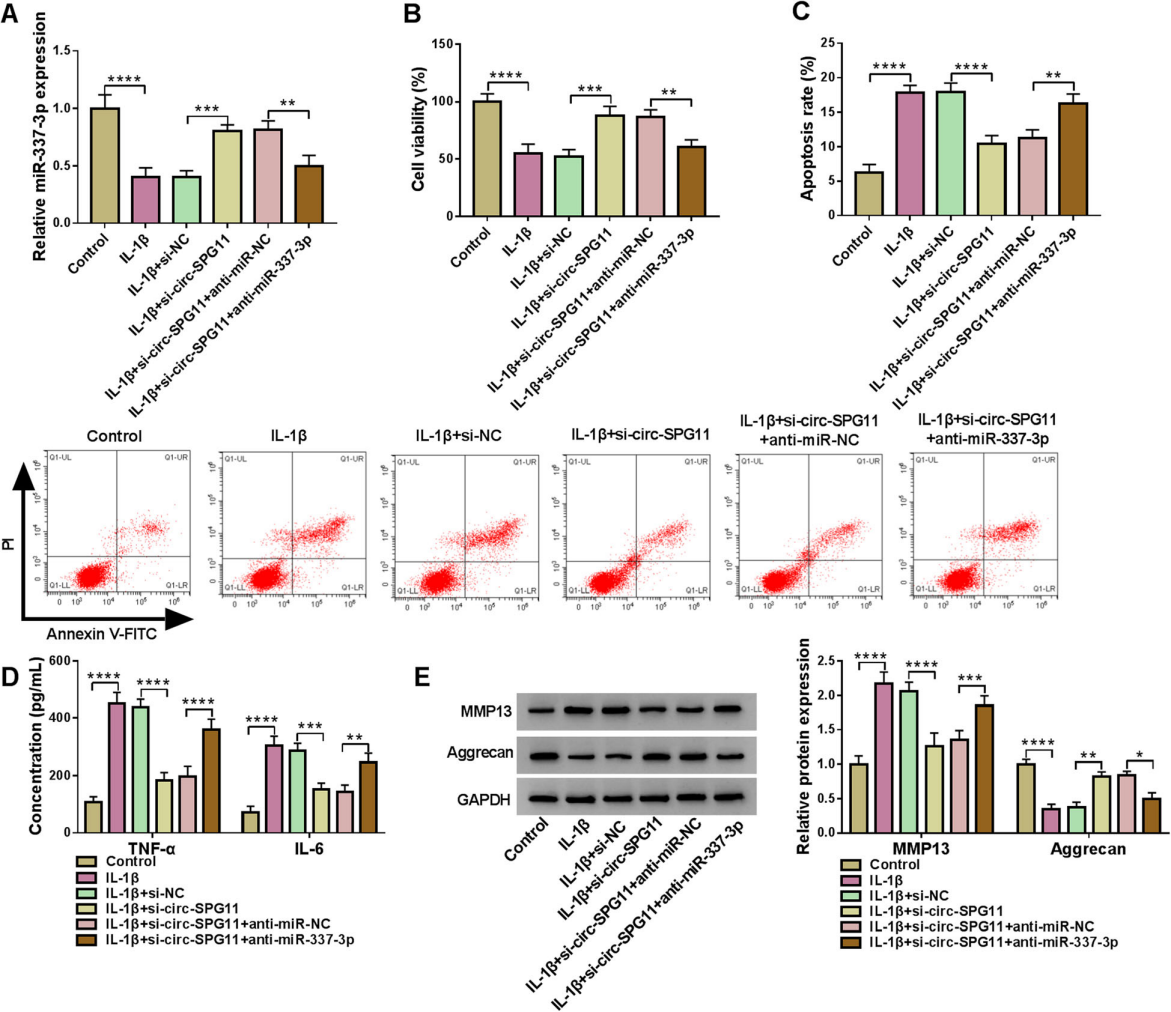
MiR-337-3p inhibition allayed si-circ-SPG11-induced promotion in proliferation, repression in apoptosis, inflammatory cytokines secretion, and ECM degradation in OA model cells. **A** MiR-337-3p expression was measured by qRT-PCR in OA cells without treatment or treated with IL-1β and transfected with si-circ-SPG11, si-circ-SPG11 + anti-miR-337-3p, or negative controls. **B** The effect of si-circ-SPG11 and anti-miR-337-3p on viability was assessed by CCK8 assay in OA cells without treatment or treated with IL-1β and transfected with si-circ-SPG11, si-circ-SPG11 + anti-miR-337-3p, or negative controls. **C** The apoptosis rate was measured by flow cytometry in OA cells without treatment or treated with IL-1β and transfected with si-circ-SPG11, si-circ-SPG11 + anti-miR-337-3p, or negative controls. **D** The role of si-circ-SPG11 and anti-miR-337-3p in inflammatory cytokines secretion was evaluated by ELISA in OA cells treated with or without IL-1β. **E** Western blot was used to detect the protein expression of MMP13 and Aggrecan in OA cells without treatment or treated with IL-1β and transfected with si-circ-SPG11, si-circ-SPG11 + anti-miR-337-3p, or negative controls. *P < 0.05, **P < 0.01, ***P < 0.001, ****P < 0.0001

To further study the functional regulatory relationship between circ-SPG11 and miR-337-3p, loss-of-function experiments were applied. Mechanically, IL-1β treatment induced the suppression in miR-337-3p expression in CHON-001 cells; meanwhile, anti-miR-337-3p reversed si-circ-SPG11-induced upregulation in miR-337-3p expression in IL-1β-treated CHON-001 cells (Fig. 5A). Furthermore, anti-miR-337-3p allayed si-circ-SPG11-induced promotion in viability in IL-1β-treated CHON-001 cells (Fig. 5B). For the apoptosis rate, si-circ-SPG11 attenuated IL-1β-induced motivation, which was further abrogated by anti-miR-337-3p in CHON-001 cells (Fig. 5C). Similarly, the inflammatory cytokines secretion was restrained by si-circ-SPG11 via interacting with miR-337-3p in IL-1β-treated CHON-001 cells (Fig. 5D). The protein expression of ECM degradation-related markers showed that anti-miR-337-3p rescued si-circ-SPG11 induced suppression in ECM degradation in IL-1β-treated CHON-001 cells (Fig. 5E). These results uncovered that circ-SPG11 sponged miR-337-3p to regulate the proliferation, apoptosis, inflammatory cytokines, and ECM degradation in OA model cells.

### MiR-337-3p targeted ADAMTS5 3′UTR

**Fig. 6:**
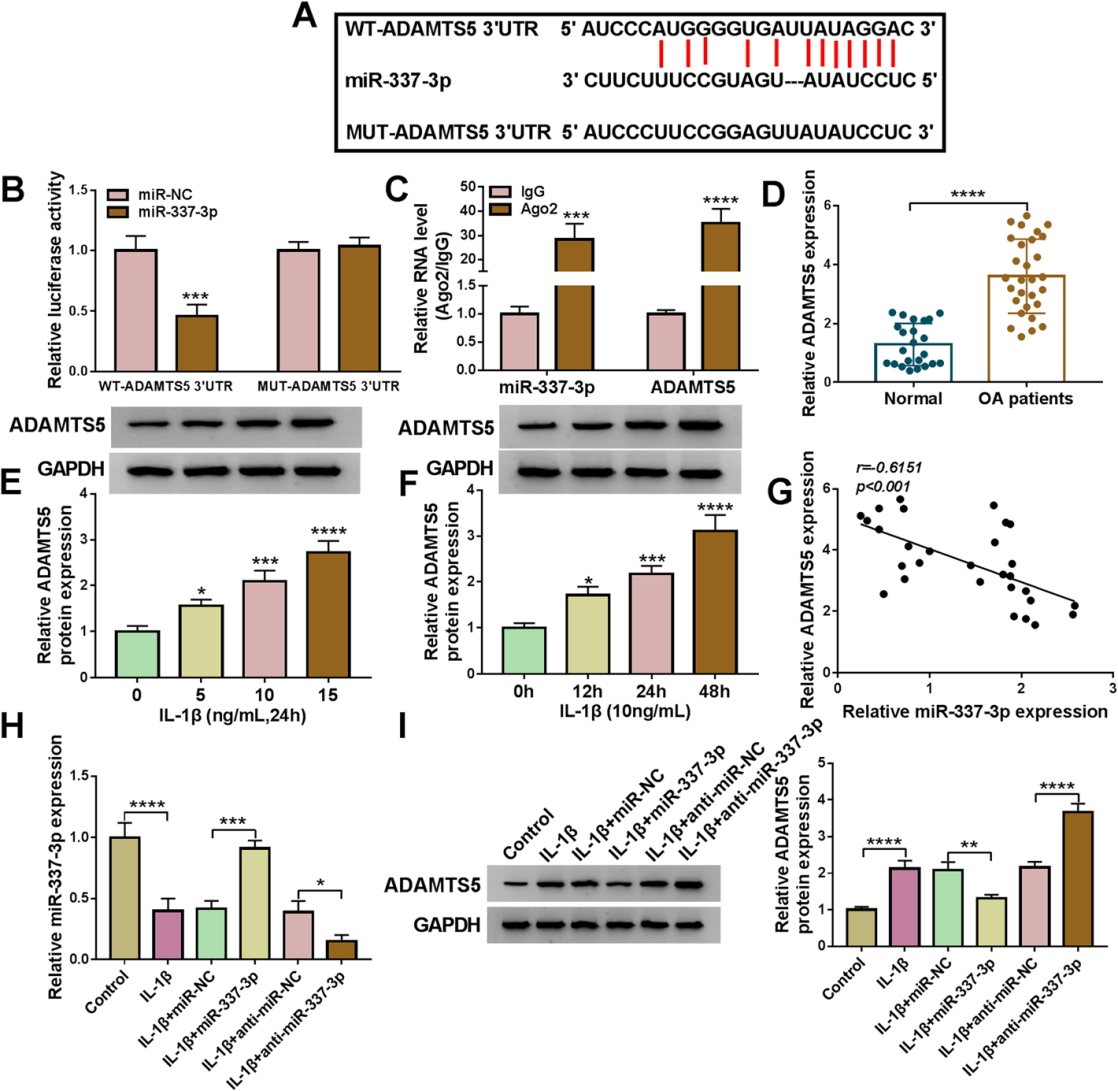
MiR-337-3p directly targeted ADAMTS5 and negatively regulated ADAMTS5 expression. **A** Starbase2.0 online database was conducted to predict the potential binding sequences between miR-337-3p and ADAMTS5 3′UTR. **B** Dual-luciferase reporter system was applied to test the combination between miR-337-3p and ADAMTS5 in OA cells. **C** RIP assay was devoted to inspecting the combination between miR-337-3p and ADAMTS5 in OA cells. **D** ADAMTS5 mRNA expression was detected by qRT-PCR in OA tissues and normal tissues. **E** ADAMTS5 protein expression was measured by western blot in CHON-001 cells treated by 0 ng/mL, 5 ng/mL, 10 ng/mL, 15 ng/mL of IL-1β for 24 h. **F** Western blot was used to test ADAMTS5 protein expression in CHON-001 cells treated by 10 ng/mL of IL-1β for 0 h, 12 h, 24 h, and 48 h. **G** Pearson correlation coefficient analyzed the relationship between ADAMTS5 mRNA expression and miR-337-3p expression. **H** MiR-337-3p expression was detected by qRT-PCR in CHON-001 cells without treatment or treated with IL-1β and transfected with miR-337-3p, anti-miR-337-3p, or negative controls. **I** ADAMTS5 protein expression was determined by western blot in CHON-001 cells without treatment or treated with IL-1β and transfected with miR-337-3p, anti-miR-337-3p, or negative controls. *P < 0.05, **P < 0.01, ***P < 0.001, ****P < 0.0001

The downstream regulatory factor of circ-SPG11/miR-337-3p was unceasingly explored to further study the role of circ-SPG11 in OA. As presented in Fig. 6A, the potential targeted sequences in ADAMTS5 3′UTR to miR-337-3p were predicted by Starbase2.0 online database. Dual-luciferase reporter system illustrated that the combination between ADAMTS5 and miR-337-3p actually existed (Fig. 6B), which was in accordance with the result of RIP assay (Fig. 6C). Clearly, a significant increase in ADAMTS5 expression was appeared in OA tissues compared with normal tissues (Fig. 6D). Meanwhile, ADAMTS5 protein expression was abundantly expressed by a dose-dependent manner and a time-dependent manner to IL-1β treatment in CHON-001 cells (Fig. 6E, F). Moreover, Pearson correlation coefficient analysis showed a negatively relationship between the expression of ADAMTS5 and miR-337-3p (Fig. 6G). In addition, miR-337-3p was downregulated in OA model cells, however, miR-337-3p was upregulated by miR-337-3p overexpression or further downregulated by anti-miR-337-3p in OA model cells (Fig. 6H). Besides, miR-337-3p could negatively regulate ADAMTS5 protein expression in OA model cells (Fig. 6I). These data elucidated that miR-337-3p directly targeted ADAMTS5 and negatively regulated ADAMTS5 expression.

### MiR-337-3p targeted ADAMTS5 to regulate the proliferation, apoptosis, inflammatory cytokines secretion, and ECM degradation in OA model cells

**Fig. 7:**
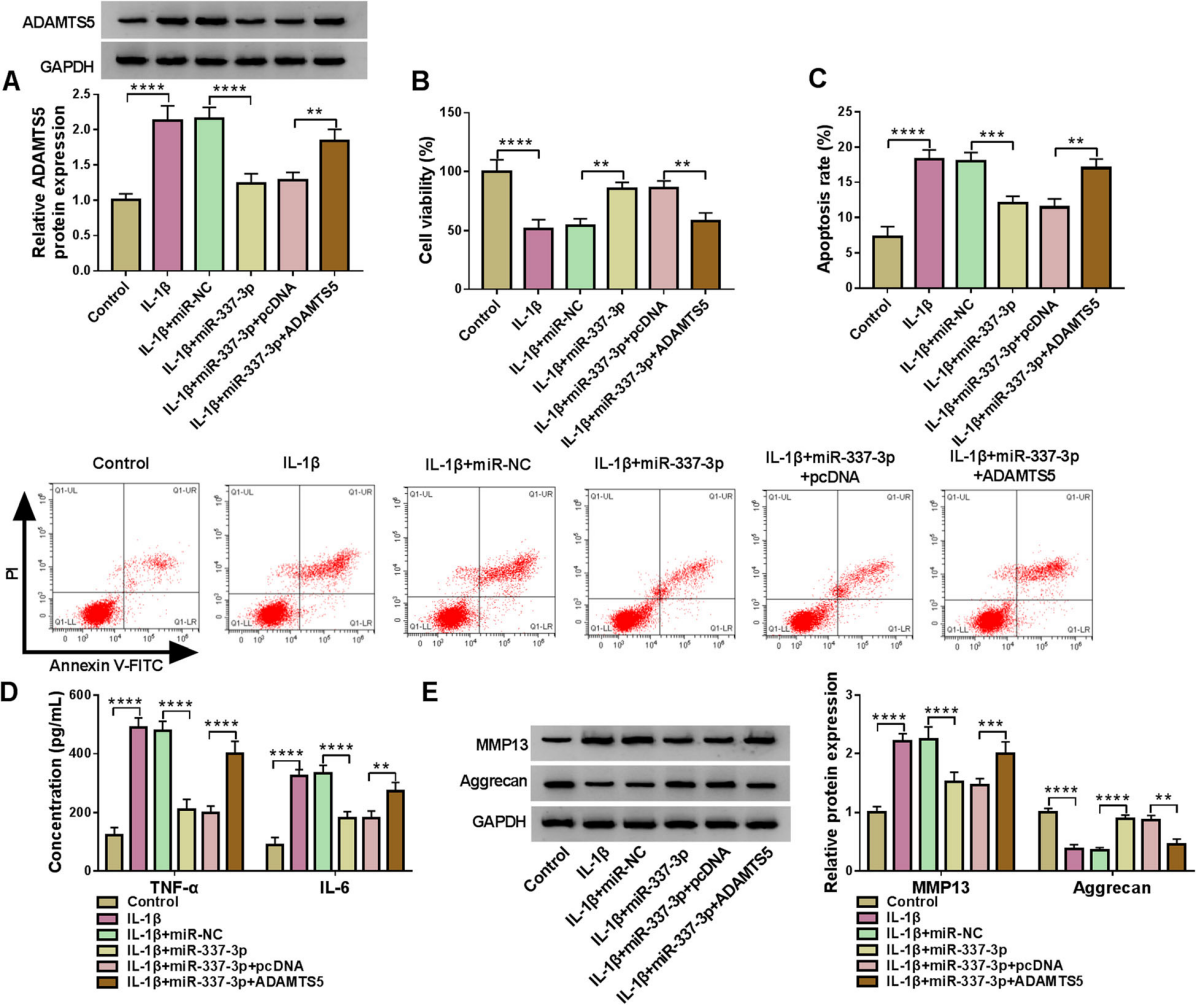
MiR-337-3p targeted ADAMTS5 to participate in OA process. **A** Western blot was employed to test ADAMTS5 protein expression in CHON-001 cells without treatment or treated with IL-1β and transfected with miR-337-3p, miR-337-3p + ADAMTS5, or negative controls. **B** CCK8 assay was devoted to determining the viability of CHON-001 cells without treatment or treated with IL-1β and transfected with miR-337-3p, miR-337-3p + ADAMTS5, or negative controls. **C** Flow cytometry assay was dedicated to monitor the apoptosis rate in CHON-001 cells without treatment or treated with IL-1β and transfected with miR-337-3p, miR-337-3p + ADAMTS5, or negative controls. **D** The generation of TNF-α and IL-6 was detected by ELISA in CHON-001 cells without treatment or treated with IL-1β and transfected with miR-337-3p, miR-337-3p + ADAMTS5, or negative controls. **E** ADAMTS5 protein expression was determined by western blot in CHON-001 cells without treatment or treated with IL-1β and transfected with miR-337-3p, miR-337-3p + ADAMTS5 or negative controls. **P < 0.01, ***P < 0.001, ****P < 0.0001.

Given the regulatory mechanism between ADAMTS5 and miR-337-3p, the functional relationship between ADAMTS5 and miR-337-3p was explored. MiR-337-3p overexpression allayed IL-1β-induced upregulation in ADAMTS5 protein expression, which was further counteracted by ADAMTS5 overexpression in CHOH-001 cells (Fig. 7A). ADAMTS5 overexpression ameliorated miR-337-3p overexpression-induced revivification in IL-1β-induced inhibition in viability in CHON-001 cells (Fig. 7B). IL-1β-induced motivation in apoptosis, inflammatory cytokines secretion, and ECM degradation was reversed by miR-337-3p overexpression, which was further allayed by ADAMTS5 overexpression in CHON-001 cells (Fig. 7C–E). These data highlighted that ADAMTS5 stimulated OA development by interacting with miR-337-3p.

### Circ-SPG11 stimulated OA progression by miR-337-3p/ADAMTS5 axis

**Fig. 8:**
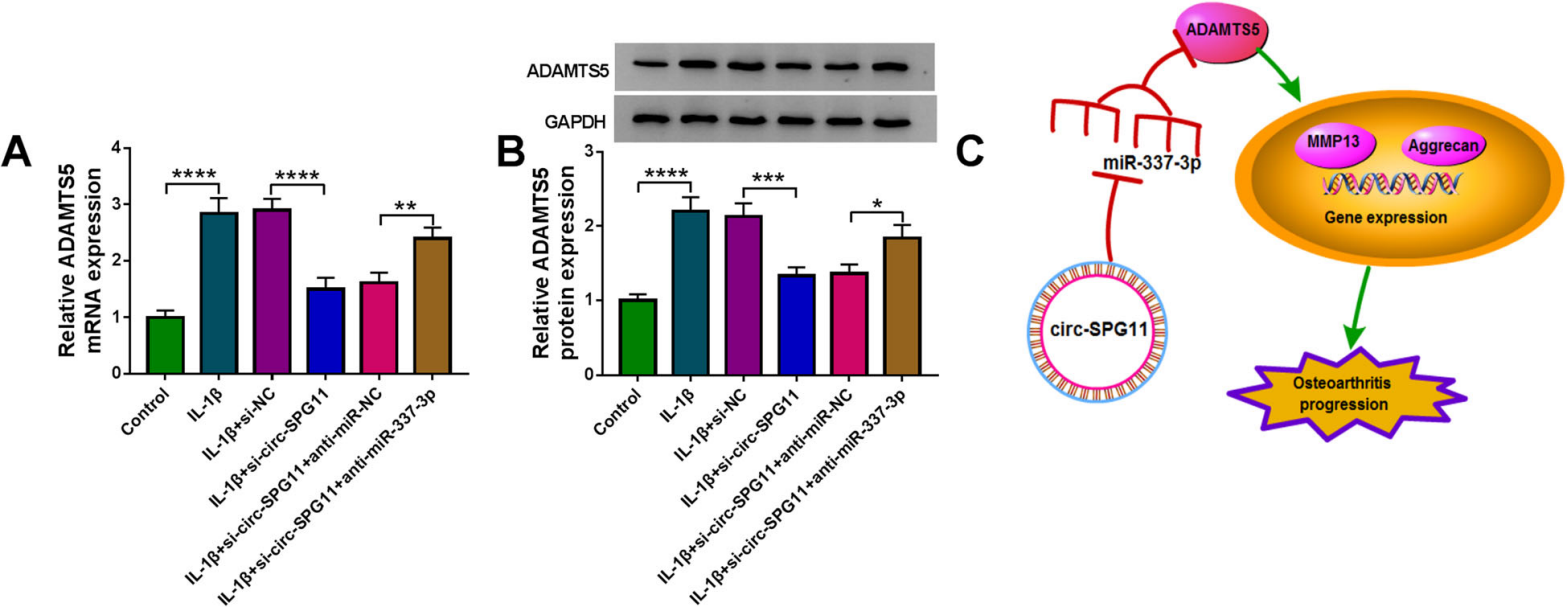
Circ-SPG11/miR-337-3p/ADAMTS5 axis was associated with OA development. **A** QRT-PCR was used to detect ADAMTS5 mRNA expression in CHON-001 cells without treatment or treated with IL-1β and transfected with si-circ-SPG11, si-circ-SPG11 + anti-miR-337-3p, or negative controls. **B** Western blot was applied to measure ADAMTS5 protein expression in CHON-001 cells without treatment or treated with IL-1β and transfected with si-circ-SPG11, si-circ-SPG11 + anti-miR-337-3p, or negative controls. **C** Schematic diagram described the signal pathway of circ-SPG11/miR-337-3p/ADAMTS5 axis in OA. *P < 0.05, **P < 0.01, ***P < 0.001, ****P < 0.0001

To perfect the regulatory mechanism, the relationship among circ-SPG11, miR-337-3p, and ADAMTS5 was further studied. ADAMTS5 mRNA or protein expression was elevated by IL-1β treatment, and this elevation was restored by si-circ-SPG11; however, anti-miR-337-3p reversed the effect of si-circ-SPG11 on IL-1β-treated CHON-001 cells (Fig. 8A, B). The schematic diagram described the circ-SPG11/miR-337-3p/ADAMTS5 pathway in regulating OA development that circ-SPG11 sponged miR-337-3p to positively regulate ADAMTS5 expression, which resulted in motivating ECM degradation, so as to stimulate OA progression (Fig. 8C). These data revealed that circ-SPG11 advanced OA development by miR-337-3p/ADAMTS5 axis.

## Discussion

OA is a time-consuming and excruciating disease for the elderly with high incidence [14]. The ECM degradation has been recognized as the primarily feature of OA [15]. The activation of chondrocytes was the inevitable result of the reaction to the dynamic alteration of the chemical and mechanical surroundings [16]. Thus, the generation of inflammatory factors (IL-1β, IL-6, and TNF-α) and the matrix-degrading enzymes (MMP13, Aggrecan) were triggered in the activated chondrocytes [17]. CircRNAs were frequently found to be associated with various diseases. Recently, Zhou et al. exposed 255 circRNAs which were differentially expressed in IL-1β-treated mouse articular chondrocytes [18]. Liu et al. found that 71 circRNAs were dysregulated in human OA tissues [19]. In this study, circ-SPG11 was abundantly expressed in OA tissues compared with normal tissues. Meanwhile, IL-1β treatment led to an increase in circ-SPG11 expression by time-dependent and dose-dependent manners in CHON-001 cells. Besides, circ-SPG11 knockdown expedited the proliferation, abated the apoptosis, inflammatory cytokines secretion, and ECM degradation in OA model cells. Has_circ_0005105 facilitated ECM degradation, inflammatory cytokines generation in IL-1β-treated chondrocytes [20]. Has_circ_0045714 promoted the apoptosis and inhibited the proliferation in chondrocytes [21].

MiRNAs always interact with circRNAs to play a bridging role in perfecting the signal pathway in diseases [22]. CircDUSP16 harbored miR-145-5p to expedite gastric cancer (GC) development [23]. CircHIPK2 sponged miR-124-2HG to activate astrocyte [24]. CircLARP4 hampered GC progression by miR-424-5p/LATS1 signal pathway [25]. CircPVT1 harbored miR-145-5p to regulate the expression of ABCC1 in chemotherapy-resistant lung cancer [26]. Analogously, circ-SPG11 directly sponged miR-337-3p to negatively regulate miR-337-3p expression. CircRNA-CDR1as harbored miR-641 to accelerate OA development [27]. CircRNA.33186 boosted OA procession by harboring miR-127-5p [28]. MiR-337-3p inhibition allayed circ-SPG11 deletion-mediated stimulation in proliferation and repression in apoptosis, inflammatory factors generation and ECM degradation in OA model cells. MiR-337-3p facilitated the proliferation and restrained the apoptosis via interacting with PTEN and AKT in OA cells [29]. Moreover, miR-337-3p directly targeted ADAMTS5 and negatively regulated ADAMTS5 expression. ADAMTS5 was targeted by miR-15a to retard the development of OA [30]. ADAMTS5 overexpression ameliorated miR-337-3p overexpression-mediated promotion in proliferation and suppression in apoptosis, inflammatory factors production, and ECM degradation in OA model cells. ADAMTS5 was the downstream factor of LncRNA MALAT1/miR-145 axis in inhibiting the proliferation and promoting the ECM degradation in OA model cells [31]. ADAMTS5 was targeted by miR-140-5p to suppress the metastasis in colorectal cancer [32]. Associatively, circ-SPG11 sponged miR-337-3p to regulate ADAMTS5 expression in OA model cells.

## Conclusion

Collectively, circ-SPG11 and ADAMTS5 were upregulated and miR-337-3p was downregulated in OA tissues and OA model cells. Circ-SPG11 knockdown promoted the proliferation and undermined the apoptosis, inflammatory factors generation, and ECM degradation in OA model cells. Meanwhile, circ-SPG11 accelerated OA development by interacting with miR-337-3p/ADAMTS5 pathway. The novel findings about the regulatory pathway of circ-SPG11 in OA might point a way for targeted therapy in OA.

## Data Availability

The analyzed datasets generated during the present study are available from the corresponding author on reasonable request.
